# True nutrient and amino acid digestibility of dog foods made with human-grade ingredients using the precision-fed cecectomized rooster assay^1^

**DOI:** 10.1093/tas/txz175

**Published:** 2019-12-06

**Authors:** Patrícia M Oba, Pamela L Utterback, Carl M Parsons, Kelly S Swanson

**Affiliations:** 1 Department of Animal Sciences, University of Illinois at Urbana-Champaign, Urbana, IL; 2 Division of Nutritional Sciences, University of Illinois at Urbana-Champaign, Urbana, IL; 3 Department of Veterinary Clinical Medicine, University of Illinois at Urbana-Champaign, Urbana, IL

**Keywords:** animal model, canine, nutrient digestion, pet food

## Abstract

For a pet diet to be labeled as human-grade, every ingredient and the finished food must be stored, handled, processed, and transported according to the current good manufacturing practices for human edible foods. Human-grade dog foods are now available and increasing in popularity, but little research has been conducted to test the digestibility of these foods. For this reason, the objective of this experiment was to determine the true nutrient and amino acid (AA) digestibilities of dog foods formulated with human-grade ingredients using the precision-fed cecectomized rooster assay. Six commercial dog foods were tested, including the Beef & Russet Potato (BRP), Chicken & White Rice (CWR), Fish & Sweet Potato (FSP), Lamb & Brown Rice (LBR), Turkey & Whole Wheat Macaroni (TWM), and Venison & Squash (VSR) formulas provided by Just Food For Dogs LLC (Irvine, CA). Before analysis, all foods were lyophilized and ground. A precision-fed rooster assay using cecectomized roosters was conducted to determine the true nutrient digestibility and standardized AA digestibilities of the foods tested. Conventional roosters were used to determine the nitrogen-corrected true metabolizable energy (TMEn) of the foods. All animal procedures were approved by the University of Illinois Institutional Animal Care and Use Committee prior to experimentation. The substrates and rooster excreta were analyzed for macronutrient and AA composition. All data were analyzed using the Mixed Models procedure of SAS (version 9.4; SAS Institute, Cary, NC). In general, all foods tested were highly digestible. Dry matter digestibility was similar among CWR, LBR, and TWR foods, and greater (*P* < 0.0001) than that of FSP and VSR foods. Organic matter digestibility was highest (*P* = 0.0002) for CWR and lowest (*P* = 0.0002) for VSR. For the majority of indispensable AA, digestibilities were greater than 85%, with some being greater than 90%. TMEn was higher (*P* < 0.0001) for BRP than the other foods, which were similar to one another. Also, TMEn values were much higher than what would be estimated by using modified Atwater factors and often above the predictive equations for metabolizable energy (ME) recommended by the National Research Council or by using Atwater factors. Although statistical differences were observed among foods, they all performed well and the foods tested had very high AA digestibilities. Additionally, the TMEn data suggest that existing methods and equations for ME prediction underestimate the energy content of the foods tested.

## INTRODUCTION

Pet owners have become progressively interested in the quality and safety of food for their animals. Consumer interests and concerns have become markedly greater since a few large-scale pet food recalls in the early 2000s (Stenske et al., 2006; Rumbeiha and Morrison, 2011; Bischoff and Rumbeiha, 2018), motivating pet owners to pursue alternative foods for their pets. Studies have shown that the majority of pets are primarily fed a commercial pet food, and most often in the form of extruded kibbles (Laflamme et al., 2008; Connolly et al., 2014; Dinallo et al., 2017). However, many pet owners have moved to pet foods having the perception of being safer or of higher quality, with claims such as “natural,” “organic,” “limited ingredients,” “human-grade,” “made in the USA,” “non-GMO,” and “clean label” being quite popular. Consumers are also demanding greater corporate transparency about the ingredient sourcing, processing, distribution, and local and wider economic and ecological impacts of pet foods, as well as company practices and values in general (Sprinkle, 2019). Strongly attached owners tend to treat their dogs as people, view their dogs as children, and/or think of themselves as pet parents (Boya et al., 2012). Because of this level of anthropomorphism, many pet owners are interested in feeding an alternative diet that they believe reinforces the human–animal bond.

To be considered a human-grade pet diet, all ingredients must be human edible and the product must be manufactured, packed, and held in accordance with federal regulations in 21 CFR 110, Current Good Manufacturing Practice in Manufacturing, Packing, or Holding Human Food, which are designed to ensure safety for human consumption and therefore can be considered edible by humans (AAFCO, 2017). Therefore, the presence of the “human-grade” term on a pet food label can only be used if the product as a whole follows the quality standards for human consumption rather than the inclusion of some ingredients that are manufactured according to the U.S. Department of Agriculture (USDA) regulations. Most of the human-grade pet foods are products that resemble “homemade” foods but are produced on a larger commercial scale.

Although human-grade dog foods are now available and increasing in popularity, little research has been conducted to test the digestibility of these foods. To our knowledge, no previous study has evaluated the protein quality and nutrient digestibility of these foods and published the results in a peer-reviewed journal. For this reason, the objective of this experiment was to determine the true nutrient and amino acid (AA) digestibilities of dog foods made with human-grade ingredients using the precision-fed cecectomized rooster assay, and to compare metabolizable energy (ME) estimate calculations (NRC; Atwater factors; modified Atwater factors) against nitrogen-corrected true metabolizable energy (TMEn) data. Our hypothesis was that these foods were of high protein quality and nutritional value that would translate into high indispensable AA digestibilities and that TMEn values would be higher than the standard ME estimate calculations.

## MATERIALS AND METHODS

### Substrates

Six cooked, complete, and balanced foods for adult dogs that were made with human-grade ingredients were tested in this study. All foods had previously passed the Association of American Feed Control Officials (AAFCO) feeding trials. Diets included the Beef & Russet Potato (BRP), Chicken & White Rice (CWR), Fish & Sweet Potato (FSP), Lamb & Brown Rice (LBR), Turkey & Whole Wheat Macaroni (TWM), and Venison & Squash (VSR) formulas manufactured and provided by Just Food For Dogs LLC (Irvine, CA). Before analysis, frozen foods were lyophilized (Dura-Dry MP microprocessor-controlled freeze-dryer; FTS Systems, Stone Ridge, NY) and ground through a 2-mm screen (Wiley mill Model 4; Thomas Scientific, Swedesboro, NJ).

### Cecectomized Rooster Assay

The protocol for the cecectomized rooster assay, including all animal housing, handling, and surgical procedures, was reviewed and approved by the Institutional Animal Care and Use Committee at the University of Illinois at Urbana-Champaign prior to experimentation. Two precision-fed rooster assays utilizing Single Comb White Leghorn roosters (1.5 to 2.5 yr old, 2.5 to 3 kg BW) were conducted as described by Parsons (1985) to determine the true nutrient digestibility and standardized AA digestibility, and TMEn content of the six foods tested. Prior to the study, cecectomy was performed on roosters under general anesthesia according to the procedures of Parsons (1985). All roosters were given at least 8 weeks to recover from surgery before being used in experiments. All birds were housed individually in cages (27.9 cm wide × 50.8 cm long × 53.3 cm high) with raised wire floors. They were kept in an environmentally controlled room (approximately 23.9°C, 17 h light:7 h dark). Before the start of the experiment, feed and water were supplied for *ad libitum* consumption.

In the first rooster assay (to determine nutrient and AA digestibility), 24 cecectomized roosters were randomly assigned to test foods (4 roosters per test substrate evaluated). In the second rooster assay (to determine TMEn), 24 conventional roosters were randomly assigned to the test foods (4 roosters per test substrate evaluated). In both assays, after 26 h of feed withdrawal and *ad libitum* water, roosters were tube-fed 20 g of the test substrates. Following crop intubation, excreta were collected for 48 h on plastic trays placed under each individual cage. Excreta samples then were lyophilized, weighed, and ground through a 0.25-mm screen prior to the analysis. Endogenous corrections for AA were made using five additional cecectomized roosters that had been fasted for 48 h. Standardized nutrient and AA digestibilities were calculated using the method described by Sibbald (1979).

### Chemical Analyses

The substrates and rooster excreta were analyzed for dry matter (DM; 105^o^C) and ash [organic matter (OM) was calculated based on ash] according to AOAC (2006). Nitrogen (N) was measured [crude protein (CP) was calculated based on N] using a Leco Nitrogen/Protein Determinator (Model FP-2000, Leco Corporation, St. Joseph, MI) according to the AOAC (2006; method 982.30E). Acid-hydrolyzed fat (AHF) concentrations were determined by acid hydrolysis according to the AACC (1983), followed by diethyl ether extraction (Budde, 1952). Total dietary fiber (TDF), including total insoluble fiber (TIF) and total soluble fiber (TSF) fractions, was determined according to Prosky et al. (1985). Gross energy (GE) was measured using a bomb calorimeter (Model 1261; Parr Instrument Com., Moline, IL). The AA was measured at the University of Missouri Experiment Station Chemical Laboratories (Columbia, MO) according to the AOAC (2006; method 982.30E).

### Nitrogen-Corrected True Metabolizable Energy (TMEn) Calculations

The calculation of TMEn was performed according to Parsons et al. (1992). The TMEn values, corrected for endogenous energy excretion using many fasted birds over many years, were calculated using the following equation:

$$\begin{array}{l} \frac{\begin{array}{c} TME_{n}\left(kcal/g\right)=EI_{fed}\;(EE_{fed}\pm 8.22\times N_{fed})\\ \pm (EE_{fasted}\pm 8.22\times N_{fasted}) \end{array}}{FI} \end{array}$$

In the above equation, EI_fed_ equals the GE intake of the test substrate consumed; EE_fed_ equals the energy in the excreta collected from fed birds; 8.22 is the correction factor for uric acid; N_fed_ equals the grams of N retained by the fed birds; EE_fasted_ equals the energy in the excreta collected from the fasted birds (16.74 kcal/g); N_fasted_ equals the g N retained by the fasted birds (1.1256 g); and FI equals the grams of dry test substrate consumed (Parsons et al., 1992).

### Metabolizable Energy Calculations

To compare against TMEn data, ME estimates were performed according to modified NRC (2006) calculations [using TDF instead of crude fiber (CF) values], Atwater factors (Atwater, 1902), and modified Atwater factors. Modified nitrogen-free extract (NFE) values (using TDF instead of CF values) and ME were calculated using the following equations:

a) NFE (%) = 100% − (% CP in DM + % AHF in DM + % TDF in DM + % Ash in DM)
b) ME (Atwater values; kcal/g) = [(4 × CP in DM) + (9 × AHF in DM) + (4 × NFE in DM)]/100
c) ME (modified Atwater values; kcal/g) = [(3.5 × CP in DM) + (8.5 × AHF in DM) + (3.5 × NFE in DM)]/100
d) ME (NRC, 2006 equation; kcal/g) =
1. Gross energy (kcal) = (5.7 × % CP in DM) + (9.4 × % AHF in DM) + [4.1 × (% NFE + % TDF in DM)]
2. Energy digestibility (%): 91.2 – (1.43 × % TDF in DM)
3. Digestible energy (DE): (kcal GE × energy digestibility) / 100
4. ME (NRC) (kcal/g) = [kcal DE – (1.04 × % CP in DM)]/100

Because the CF assay does not accurately measure fiber, it should not be used for estimating the fiber content in pet foods (Fahey et al., 2019). Therefore, the TDF assay, which allows the measurement of both soluble and insoluble fiber fractions and is a much better fiber estimate, was used in the equations above. Using TDF to estimate NFE has been shown to have a high correlation with starch content in dog foods (de-Oliveira et al., 2012).

### Statistical Analyses

All data were analyzed using the Mixed Models procedure of SAS (version 9.4; SAS Institute, Cary, NC). Substrates were considered to be a fixed effect, and roosters were considered to be a random effect. Tukey’s multiple comparison analysis was used to compare least square means for experiment-wise error. Differences were considered significant with *P* < 0.05.

## RESULTS

### Chemical Composition

The chemical composition of test foods is presented in Table 1. Of note, the DM contents listed for the foods were the values present after the freeze-drying process, which was needed to properly conduct the chemical analyses and dosing for the rooster experiments. All other nutrients are represented on a dry matter basis (DMB). Ash content was highest in FSP (7.4%) and lowest in CWR (3.82%). The VSR diet (47.6% DMB) had the highest CP concentration, while LBR (24.0% DMB) was the lowest. The BRP diet (46.4% DMB) had the highest AHF concentration, while CWR, FSP, TWM, and VSR had similar AHF content (16.5% to 18.0% DMB). The BRP diet had the lowest TDF, TIF, and TSF concentrations (4.2%, 3.9%, and 0.3% DMB, respectively), while VSR had the highest TDF, TIF, and TSF concentrations (14.5%, 9.3%, and 5.2% DMB, respectively). The CWR diet had the highest NFE (43.9% DMB), while the BRP and VSR foods had the lowest NFE (14.1% and 14.8% DMB, respectively). The CWR diet had the lowest GE (5.19 kcal/g DM), while BRP had the highest (6.69 kcal/g DM). Concentrations of indispensable and dispensable AA are presented in Table 2. Sweet potatoes were used in the BRP, FSP, and VSR recipes; russet potatoes and green beans were used in the BRP and FSP recipes; carrots were used in the BRP, CWR, LBR, and TWM recipes; apples were used in the BRP and CWR recipes; spinach was used in the CWR and LBR recipes; broccoli was used in the FSP and TWM recipes; and cranberries were used in the TWM and VSR recipes. Safflower oil was used in the BRP, FSP, LBR, and VSR recipes, and Icelandic premium EPA and DHA were used in all recipes except for the FSP recipe. The protein sources were different for all foods tested.

**Table 1.** Chemical composition and true macronutrient digestibility of dog foods made with human-grade ingredients using the precision-fed cecectomized rooster assay^1^

Item	BRP^2^	CWR^3^	FSP^4^	LBR^5^	TWM^6^	VSR^7^	SEM	*P*-value
Chemical composition								
DM^8^, %	92.89	93.92	90.82	95.18	97.38	93.88	—	—
--------------------Dry matter basis --------------------								
Ash, %	4.16	3.82	7.39	3.99	3.92	6.61	—	—
CP, %	31.14	29.10	38.89	24.08	34.54	47.58	—-	—
AHF, %	46.40	17.27	17.43	27.02	17.98	16.50	—	—
TDF, %	4.22	5.92	10.12	7.16	12.48	14.54	—	—
TIF,%	3.91	4.45	7.96	4.05	8.76	9.31	—	—
TSF,%	0.30	1.47	2.16	3.11	3.72	5.23	—	—
NFE, %^9^	14.09	43.90	26.18	37.74	31.07	14.77	—	—
GE, kcal/g	6.69	5.19	5.37	5.63	5.40	5.44	—	—
Nutrient digestibility								
DM, %	74.0^ab^	82.3^a^	67.2^b^	81.0^a^	78.0^a^	67.6^b^	2.210	0.0002
OM, %	81.9^bc^	89.2^a^	76.4^cd^	87.3^ab^	81.9^bc^	74.1^d^	1.404	<0.0001
AHF, %	87.9^b^	94.6^a^	92.7^a^	85.0^b^	93.1^a^	93.1^a^	0.829	<0.0001
GE, %	86.9^bc^	93.7^a^	83.8^c^	89.1^b^	86.6^bcd^	82.3^d^	0.911	<0.0001
Metabolizable energy estimates								
TMEn, kcal/g	5.83^a^	4.75^b^	4.71^b^	4.89^b^	4.71^b^	4.62^b^	0.088	<0.0001
ME (Atwater values)^10^, kcal/g	5.99	4.47	4.17	4.90	4.24	3.98	—	—
ME (modified Atwater values)^11^, kcal/g	5.53	4.02	3.76	4.46	3.83	3.58	—	—
ME (NRC equation)^12^, kcal/g	5.83	4.37	4.06	4.63	3.96	3.80	—	—
TMEn/GE, %	87.1	91.5	87.8	86.8	87.2	84.9	1.547	0.1350

^1^ *n* = 4 roosters per treatment.

^2^BRP = Beef & Russet Potato (ingredients: ground beef, russet potatoes, sweet potatoes, green beans, carrots, safflower oil, beef liver, green peas, apples, Icelandic premium EPA and DHA, natural calcium, phosphorus amino acid chelate, magnesium bisglycinate chelate, taurine, choline chloride, natural kelp, vitamin E, biotin, selenium amino acid chelate, manganese bisglycinate chelate, zinc oxide, vitamin D3, vitamin B1, riboflavin).

^3^CWR = Chicken & White Rice (ingredients: chicken thigh, long grain white rice, spinach, carrots, apples, chicken gizzard, chicken liver, Icelandic premium EPA and DHA, calcium pyrophosphate, natural calcium, choline bitartrate, natural kelp, magnesium bisglycinate chelate, iron bisglycinate chelate, copper bisglycinate chelate, vitamin D3, vitamin B12, riboflavin).

^4^FSP = Fish & Sweet Potato (ingredients: Pacific cod, sweet potatoes, russet potatoes, green beans, broccoli, safflower oil, natural calcium, phosphorus amino acid chelate, natural kelp, choline chloride, vitamin E, iron bisglycinate chelate, zinc oxide, biotin, copper citrate, riboflavin, vitamin B12).

^5^LBR = Lamb & Brown Rice (ingredients: ground lamb, long grain brown rice, cauliflower, carrots, lamb liver, spinach, blueberries, safflower oil, Icelandic premium EPA and DHA, natural calcium, phosphorus amino acid chelate, choline bitartrate, potassium chloride, natural kelp, sodium chloride, vitamin E, iron citrate, selenium amino acid chelate, zinc oxide, vitamin D3, riboflavin).

^6^TWM = Turkey & Whole Wheat Macaroni (ingredients: ground turkey, whole wheat macaroni, broccoli, zucchini, carrots, turkey liver, cranberries, premium EPA and DHA, natural calcium, phosphorus amino acid chelate, choline bitartrate, potassium chloride, natural kelp, sodium chloride, taurine, vitamin E, magnesium bisglycinate chelate, zinc oxide, copper bisglycinate chelate, manganese gluconate, vitamin D3, riboflavin, vitamin B12, vitamin B1).

^7^VSR = Venison & Squash (ingredients: venison, butternut squash, sweet potatoes, brussel sprouts, cranberries, safflower oil, premium EPA and DHA, natural calcium, phosphorus amino acid chelate, choline bitartrate, potassium chloride, natural kelp, sodium chloride, taurine, vitamin E, magnesium bisglycinate chelate, zinc oxide, copper bisglycinate chelate, manganese gluconate, vitamin D3, riboflavin, vitamin B12, vitamin B1).

^8^DM = dry matter, OM = organic matter, CP = crude protein, AHF = acid-hydrolyzed fat, TDF = total dietary fiber, TIF = total insoluble fiber, TSF = total soluble fiber, NFE = nitrogen-free extract, GE = gross energy, N = nitrogen, TMEn = nitrogen-corrected true metabolizable energy, ME = metabolizable energy.

^9^NFE = 100% − (% CP in DM + % AHF in DM + % TDF in DM + % Ash in DM).

^10^ME (Atwater values; kcal/g) = [(4 × CP in DM) + (9 × AHF in DM) + (4 × NFE in DM)]/100.

^11^ME (modified Atwater values; kcal/g) = [(3.5 × CP in DM) + (8.5 × AHF in DM) + (3.5 × NFE in DM)]/100.

^12^ME (NRC, 2006 equation):

-Gross energy (GE) (kcal) = (5.7 × % CP in DM) + (9.4 × % AHF in DM) + [4.1 × (% NFE + % TDF in DM)]

-Energy digestibility (ED) (%): 91.2 – (1.43 × % TDF in DM)

-Digestible energy (DE): (kcal GE × ED)/100

-ME (NRC) (kcal/g) = [kcal DE – (1.04 × % CP in DM)]/100

^a–d^Within a row, means lacking a common superscript differ (*P* < 0.05); *n* = 4 roosters per treatment.

**Table 2.** Indispensable and dispensable amino acid (AA) concentrations (% DM) of dog foods made with human-grade ingredients

Item	BRP^*1*^	CWR	FSP	LBR	TWM	VSR
Indispensable AA						
Arginine	1.75	1.81	2.31	1.41	1.99	2.75
Histidine	0.88	0.77	0.81	0.59	0.87	1.30
Isoleucine	1.27	1.33	1.85	0.97	1.55	1.88
Leucine	2.12	2.20	3.00	1.70	2.57	3.26
Lysine	2.32	2.20	3.45	1.61	2.31	3.48
Methionine	0.67	0.73	1.14	0.54	0.79	1.00
Phenylalanine	1.12	1.17	1.54	0.92	1.43	1.73
Threonine	1.19	1.18	1.67	0.93	1.32	1.82
Tryptophan	0.30	0.34	0.48	0.25	0.35	0.44
Valine	1.40	1.44	2.03	1.15	1.63	2.18
Selected dispensable AA						
Alanine	1.67	1.60	2.18	1.31	1.74	2.72
Aspartic acid	2.70	2.52	4.23	1.99	2.63	4.08
Cysteine	0.33	0.36	0.43	0.33	0.48	0.48
Glutamic acid	3.77	4.11	5.86	3.15	6.29	5.86
Glycine	1.85	1.51	1.75	1.31	1.68	3.08
Proline	1.26	1.15	1.26	0.99	1.87	2.20
Serine	0.97	1.00	1.50	0.81	1.23	1.49
Tyrosine	0.98	1.11	1.34	0.82	1.12	1.54
Taurine	0.13	0.33	0.34	0.24	0.47	0.24

^*1*^BRP = Beef & Russet Potato, CWR = Chicken & White Rice, FSP = Fish & Sweet Potato, LBR = Lamb & Brown Rice, TWM = Turkey & Whole Wheat Macaroni, VSR = Venison & Squash.

### Cecectomized Rooster Assay

True DM digestibility was similar among CWR, LBR, and TWM (78.0% to 82.3%), and greater (*P* = 0.0002) than FSP and VSR (67.2% to 67.6%). True OM digestibility was similar for CWR and LBR, but was higher (*P* < 0.0001) for CWR (89.2%) than BRP, FSP, TWM, and VSR. Furthermore, true OM digestibility was higher (*P* < 0.0001) for BRP and TWM than VSR, which had the lowest (74.1%). True AHF digestibility was similar among CWR, FSP, TWM, and VSR (92.7% to 94.6%), and greater (*P* < 0.0001) than BRP and LBR (85.0% to 87.9%). True GE digestibility was highest for CWR (93.7%), which was higher (*P* < 0.0001) than the other treatments. The second highest true GE digestibility values were for LBR (89.1%), which was higher (*P* < 0.0001) than BRP (86.9%), TWM (86.6%), and FSP (83.8%), which were higher (*P* < 0.0001) than that for VSR (82.3%) that was lowest.

TMEn values were higher (*P* < 0.0001) for BRP (5.83 kcal/g) than all other foods tested (4.62 kcal/g to 4.89 kcal/g). However, the TMEn expressed as a percentage of GE were similar among all foods (84.9 to 91.5), with no statistical differences observed (Table 1). As shown in Table 1, all equations for estimating ME content, including the use of Atwater factors, modified Atwater factors, and the NRC (2006) equation underestimated the energy content of the CWR, FSP, TWM, and VSR foods. For the LBR diet, the best estimate was the use of Atwater factors. For the BRP diet, the NRC equation was the best estimate.

Standardized AA digestibility data are presented in Table 3. For the majority of the foods, all indispensable AA digestibilities were greater than 80%, with the exception of threonine in LBR and TWM (79.0% and 79.7%, respectively). For lysine, the FSP diet (90.1%) had a higher (*P* = 0.0362) digestibility than TWM (86.3%). For methionine, the LBR diet (87.3%) had a lower (*P* = 0.0021) digestibility than FSP and VSR (93.4% and 92.7%, respectively). For tryptophan, the LBR diet (89.3%) had a lower (*P* = 0.0117) digestibility than that FSP and VSR (93.4% and 92.7%, respectively).

**Table 3.** True amino acid (AA) digestibilities (%) of dog foods made with human-grade ingredients using the precision-fed cecectomized rooster assay^1^

AA	BRP^2^	CWR	FSP	LBR	TWM	VSR	SEM	*P*-value
Indispensable AA								
Arginine	88.3	89.7	89.3	88.8	88.3	90.1	1.046	0.7636
Histidine	85.0	87.7	84.2	84.5	85.9	85.7	1.706	0.7528
Isoleucine	86.7	87.8	88.3	85.1	86.6	87.9	1.054	0.3388
Leucine	87.8	88.3	89.2	86.3	87.6	89.4	0.979	0.3115
Lysine	86.7^ab^	90.1^ab^	90.1^a^	87.2^ab^	86.3^b^	87.8^ab^	0.954	0.0362
Methionine	90.2^ab^	90.2^ab^	92.7^a^	87.3^b^	89.7^ab^	91.1^a^	0.736	0.0021
Phenylalanine	86.4	87.0	87.0	85.2	86.9	88.1	1.020	0.5228
Threonine	80.4	82.1	83.4	79.0	79.7	83.6	1.546	0.2088
Tryptophan	90.9^ab^	91.4^ab^	93.4^a^	89.3^b^	90.4^ab^	92.7^a^	0.739	0.0117
Valine	83.9	85.1	86.0	83.0	83.1	86.3	1.311	0.3520
Selected dispensable AA								
Alanine	86.8	87.2	87.1	85.3	84.8	88.2	0.968	0.1923
Aspartic acid	85.6^ab^	85.4^ab^	87.4^a^	83.7^ab^	82.4^b^	86.6^ab^	0.976	0.0204
Cysteine	56.1	59.0	60.8	60.9	59.6	58.5	2.937	0.8664
Glutamic acid	84.6^ab^	86.6^ab^	89.0^ab^	84.2^b^	89.8^a^	86.8^ab^	1.148	0.0160
Glycine	44.7^a^	38.1^ab^	34.5^ab^	22.1^b^	34.1^ab^	47.3^a^	3.748	0.0024
Proline	82.0^abc^	81.3^abc^	79.3^c^	79.9^bc^	85.6^ab^	85.8^a^	1.303	0.0069
Serine	76.3	78.3	81.8	76.9	77.6	80.1	1.977	0.3847
Tyrosine	82.1	85.1	85.3	81.8	82.7	83.7	1.094	0.1499

^1^ *n* = 4 roosters per treatment.

^2^BRP = Beef & Russet Potato, CWR = Chicken & White Rice, FSP = Fish & Sweet Potato, LBR = Lamb & Brown Rice, TWM = Turkey & Whole Wheat Macaroni, VSR = Venison & Squash.

^a–c^Within a row, means lacking a common superscript differ (*P* < 0.05).

For all foods, the cysteine (60.9% to 56.1%) and glycine (47.3% to 22.1%) digestibilities were lower than 80%. Proline was lower than 80% for FSP and LBR (79.3% and 79.9%, respectively), and serine was lower than 80% for the majority of the foods, with the exception of FSP and VSR (81.8% and 80.1%, respectively). The FSP diet (87.4%) had a higher (*P* = 0.0204) digestibility of aspartic acid than TWM (92.4%). TWM (89.8%) had a higher digestibility of glutamic acid than LBR (84.2%). Glycine digestibility was lower for LBR (22.1%) in comparison to BRP and VSR (44.7% and 47.3%, respectively). Proline digestibility was higher for VSR (85.8%) than FSP (79.3%).

## DISCUSSION

Pet owners have become more interested in human-grade ingredients and foods recently (Sprinkle, 2019). As a result, pet food companies are considering the feasibility of developing human-grade foods and claiming it on their product labels (Dzanis, 2017). Few studies have evaluated the quality of human-grade foods or ingredients and published the results in peer-reviewed journals.

The cecectomized rooster assay or ileal‐cannulated animals are recommended to evaluate the digestibility of ingredients or foods because it provides data with less influence of gut microbiota in the large intestine. In general, the true nutrient and AA digestibility values are lower than the apparent fecal nutrient digestibility values because they do not include losses from the fermentation by gut microbiota in the large intestine. This has been demonstrated by several research groups. First, a study comparing the apparent total tract and ileal digestibility assays in ileal-cannulated dogs reported that apparent total tract digestibility values for DM, OM, and CP were higher compared with ileal digestibility values. Those researchers also reported that ileal digestibilities for most AA, except for methionine, isoleucine, lysine, phenylalanine, and alanine, were lower if measured at the ileum (Hendriks et al., 2013). Furthermore, a study using six ileal-cannulated dogs (*n* = 6/group; 6 × 6 Latin square design) and 24 cecectomized roosters (*n* = 4/group; completely randomized design) compared the digestibility of six animal byproduct foods and reported high correlations (*r* = 0.90 for total essential AA; *r* = 0.92 for total AA) between rooster and dog data (Johnson et al., 1998). Because the total tract digestibility assay in dogs can overestimate the digestibility of dietary AA and CP, it is not an accurate method for the measurement of absorption (Hendriks et al., 2013). For this reason, a direct comparison of values of true nutrient digestibility and apparent nutrient digestibility is not recommended.

A study evaluating commercial dry dog foods that exceeded the nutrient recommendations as set by AAFCO for an adult maintenance diet found that the ileal digestibility for DM ranged from 64.4% to 80.7% (average of 75.1%), OM ranged from 69.5% to 85.4% (average of 79.4%), and AHF ranged from 93.9% to 98.2% (average of 92.4%) (Hendriks et al., 2013). In the present study, DM digestibility ranged from 60.8% to 86.3% (average of 75.0%), OM ranged from 72.8% to 92.9% (average of 81.8%), and AHF ranged from 82.6% to 96.3% (average of 91.1%). Therefore, foods made with human-grade ingredients were comparable to dry dog foods that exceeded the nutrient recommendations as set by AAFCO for an adult maintenance diet when it came to overall macronutrient content.

A previous study reported that DM digestibility coefficients were lower for plant protein-based foods (main protein sources included corn gluten feed, corn gluten meal, and soybean meal, 28.5% CP DMB) (71.2%) than animal protein-based foods (chicken and chicken meal as the main protein sources, 34.5% CP DMB; 83.7%) using ileal‐cannulated dogs (Dikeman et al., 2007). Another study tested soybean-based ingredients that are traditionally used in pet foods in ileal-cannulated dogs. In that study, the small intestinal DM digestibility did not differ among foods, with digestibilities ranging from 74.0% to 80.9% (Yamka et al., 2005) and similar to that of the present study. Clapper et al. (2001) tested soy protein isolates and soy protein concentrates in ileal-cannulated dogs. Such proteins undergo processing that enhances the protein fraction, resulting in high-quality ingredients that are typically used in human foods and supplements. In that study, apparent ileal digestibilities of DM (ranged from 70.0% to 78.4%), OM (ranged from 75.1% to 81.4%), and AHF (ranged from 93.9% to 95.9%) were not different among treatments (Clapper et al., 2001). Another study used ileal-cannulated dogs to compare human-grade cuts of beef loin, pork loin, chicken breast, pollock fillet, and salmon fillet that were processed to minimize degradation prior to extrusion (Faber et al., 2010). In that study, digestibilities were extremely high, with DM digestibility ranging from 86.1% to 87.4%, OM digestibility ranging from 89.0% to 89.9%, and AHF digestibility ranging from 97.0% to 97.6% (Faber et al., 2010). Therefore, the DM, OM, and AHF digestibilities of dry foods using traditional ingredients were inferior or similar to cooked foods using human-grade ingredients depending on the source of protein used.

While macronutrient digestibility is important, the small intestinal AA digestibility data are the most important from the nutritional requirement standpoint. These data are impossible to derive from the total tract (fecal) sampling and directly correlate with the protein quality of the diet. Taking this information into account, the indispensable AA ileal digestibilities from the dry dog food reported by Hendriks et al. (2013) was, in general, numerically lower than the indispensable AA digestibilities reported in the present study. The indispensable AA digestibility for all soybean foods tested by Yamka et al., (2005) was all lower (ranged from 54.8% to 82.6%) than the foods made with human-grade ingredients (ranged from 79.0% to 93.4%) tested in the present study. However, the AA digestibilities for arginine, histidine, isoleucine, leucine, and lysine of high-quality soy protein isolates and concentrates (ranged from 86.0% to 94.3%) were high and more similar to the values reported in the current study (Clapper et al., 2001). These data demonstrate that both animal- and plant-based foods may have very high protein digestibilities and quality, but depends on raw materials and processing.

Several factors may affect the digestibility of a diet, including the ingredient source and its chemical composition. In previous studies, an increase in ash content resulted in a reduction in most essential AA and sometimes digestibility (Partanen, 1994; Johnson et al., 1998; Shirley and Parsons, 2001; Cramer et al., 2007). Most of the protein present in bone is composed of collagen, which is deficient in most essential AA, especially tryptophan (Eastoe and Long, 1960) and is difficult to digest. Additionally, pork crackling (33.5% collagen; 25.7% elastin) has lower biological values of protein compared to pork tenderloin (2.7% collagen; 1.8% elastin), because it contains a substantial proportion of connective tissues such as collagen and elastin fibers (Mitchell et al., 1927). Therefore, high bone and/or connective tissue concentrations may negatively affect the AA profile and digestibility. The ash content of all foods tested in this study was relatively low (3.8% to 7.4%) and likely did not affect the results.

Dietary fiber content, however, was quite variable in the current study (4.2% to 14.5% TDF). The amount and type of dietary fiber are known to affect nutrient digestibility. A study comparing insoluble dietary fiber (wood cellulose; 6.8% TDF, 6.8% TIF, and 0% TSF DMB) and soluble dietary fiber (mixture of whole oat flour, toasted oats, tomato pomace, fresh potato, and mixed dehydrated vegetables; 10.3% TDF, 6.9% TIF, and 3.5% TSF DMB) reported that the insoluble fiber (88.7%) treatment had a higher DM digestibility coefficient than the soluble fiber treatment (83.8%) (Dikeman et al., 2007). Although the amount of fiber did not appear to negatively influence AA digestibilities, the fiber content may have impacted the DM and OM digestibilities. Although many try to maximize nutrient digestibility, the addition of fiber, as done in the foods tested in this study, may support gastrointestinal health and the overall health of the pet (McRorie and Fahey, 2015). Therefore, the inclusion of fiber is recommended and beneficial for the animal, as long as the AA digestibility remains high, as in the foods tested in the present study.

The calculations used to estimate ME is a point of contention among pet food manufacturers, especially those of high digestibility. For human foods, the Atwater factors of 4, 9, and 4 kcal/g for digestible carbohydrate, fat, and protein, respectively, are commonly used to estimate ME (Atwater, 1902). Those factors were calculated using estimated digestibility coefficients of 96% for fat and carbohydrate and 91% for protein (Harris, 1966). The Atwater factors are recommended for estimating ME of homemade dog and cat foods and for commercial products having very high digestibility coefficients (NRC, 2006). Because the majority of pet foods in the past had lower digestibility than that of human foods, Atwater factors overestimated energy content for most pet foods. Because of this, the NRC (1985) suggested that digestibility coefficients of 80%, 90%, and 85% for protein, fat, and digestible carbohydrate, respectively, were appropriate for commercial dog foods. Those estimates led to the use of modified Atwater factors (3.5, 8.5, and 3.5 kcal/g for protein, fat, and digestible carbohydrate, respectively). Although those values provide a better estimate of ME for pet foods than do the Atwater factors, they underestimate the energy content of highly digestible foods, including today’s premium or super-premium diets (Laflamme, 2001; Asaro et al., 2017).

To our knowledge, there are no equations that have tested the accuracy of ME estimates for pet foods using human-grade ingredients. In the present study, the true AHF digestibility was over 90% for all foods with the exception of BRP and LBR (87.9% and 85.0%, respectively). Additionally, all ME estimates using Atwater factors modified Atwater factors, and the NRC equation underestimated the energy content for all foods with high AHF digestibility (CWR, FSP, TWM, and VSR). For the foods having a lower AHF digestibility (LBR and BRP), ME estimates using the Atwater factors had similar TMEn values (5.99 vs. 5.83 and 4.9 vs. 4.89 kcal/g, respectively), while the NRC equation had the same ME as the TMEn calculated for the BRP diet. For this reason, the use of Atwater factors for ME estimation of foods made with human-grade ingredients may only be accurate if the true AHF digestibility is lower than 90%.

Although statistical differences were observed among foods tested in this study, all foods performed very well. All foods tested had very high AA digestibilities, with most exceeding 85% and some over 90%. Additionally, the TMEn data suggest that the predictive equations for ME recommended by NRC (2006), Atwater factors, or modified Atwater factors underestimate the energy content for most foods tested so that their use for pet foods made with human-grade ingredients is questionable.
